# Infection prevention practices and associated factors among healthcare professionals in West Gojjam Zone public Hospitals Northwest Ethiopia, 2023

**DOI:** 10.1371/journal.pone.0338621

**Published:** 2026-01-30

**Authors:** Muluken Kindu, Muluken Azage Yenesew, Genet Gedamu Kassie

**Affiliations:** 1 East Gojam Zone Health Department, Ethiopia; 2 Department of Environmental Health, School of Public Health, College of Medicine and Health Sciences, Bahir Dar University, Bahir Dar, Ethiopia; ICDDRB: International Centre for Diarrhoeal Disease Research Bangladesh, BANGLADESH

## Abstract

**Background:**

Inadequate adherence to infection prevention and control standards places millions of patients and healthcare workers at risk of infectious diseases worldwide, including healthcare acquired infections. Effective infection prevention and control measures and interventions have been done after the occurrence of the COVID-19 pandemic; however, there is no data that shows infection prevention and control practice of healthcare professionals in West Gojjam zone hospitals.

**Objective:**

To assess infection prevention practice and associated factors among healthcare professionals in West Gojjam Zone public hospitals.

**Methods:**

A mixed institutional-based cross-sectional study was conducted among healthcare professionals in West Gojjam Zone public hospitals from March 10 to April 10, 2023. A simple random sampling technique was used to select 454 participants. A structured self-administered questionnaire, key informant interview guide, and observational checklist were used to gather the information. The collected data was entered into Epi-data 4.6 and exported into SPSS version 27 for analysis. For quantitative data, bivariate and multivariate generalized estimating equation analysis was computed, considering p < 0.05 to be statistically significant at the final model. The qualitative data was transcribed, translated, coded, and analyzed thematically. Finally, the qualitative data triangulated with quantitative data.

**Result:**

Four hundred thirty-four (95.6%) healthcare professionals participated in the study. The overall infection prevention practice of healthcare professionals in West Gojjam zone hospitals was 32.7% (95% CI: 28.29%−37.15%). Knowledge of participants (1.95, 95% CI: 1.19–3.19), attitude (AOR = 1.96, 95%CI: 1.24–3.12), profession of midwives (AOR = 0.467, 95%CI: 0.24–0.92), and working in Adet, Dembecha, Durbete, Feresbet, Finote Selam, and Liben hospitals, respectively, were the significant factors of infection prevention practice and training. Infrastructure, budget, supplies, and attitude were the challenges of infection prevention supplemented by the qualitative data.

**Conclusion:**

The study revealed that overall infection prevention practice was poor. Participant’s knowledge, participant’s attitude, and participant’s profession were significant factors for infection prevention practice, and there was variation in infection prevention practice between hospitals. The identified modifiable factors are the area of intervention to improve infection prevention practices.

## Introduction

Infection prevention and control (IPC) refers to a set of evidence-based practices aimed at preventing healthcare-associated infections (HAIs) and protecting both patients and health workers from avoidable harm [1,2]. Strong IPC programs are essential for improving care quality, reducing HAIs, combating antimicrobial resistance (AMR), and managing emerging infectious threats, which are critical components of universal health coverage [3]. HAIs are among the most common adverse events in healthcare worldwide, affecting hundreds of millions of patients annually in both developed and developing countries [4].According to the Global Report on Infection Prevention and Control, effective IPC measures can reduce the risk of HAIs by up to 70% [5]. In Ethiopia, IPC is a national priority, encompassing hand hygiene, use of personal protective equipment, instrument processing, safe injection practices, and healthcare waste management [2]. Despite these efforts, poor adherence to IPC protocols remains a major concern, putting healthcare workers and patients at risk. Contributing factors include inadequate infrastructure, limited resources, lack of training, and poor knowledge of IPC standards [6]. The COVID-19 pandemic further exposed gaps in IPC policy, coordination, and supply chains, particularly in low-resource settings [7].

A recent systematic review in Ethiopia showed that only 52.2% of healthcare professionals reported safe IPC practices, with variations by region and profession [8]. The Ethiopian Ministry of Health has updated its IPC guidelines to address current challenges, incorporating new topics such as AMR, outbreak management, and revised disinfection protocol [2,9]. Previous studies in Ethiopia have limitations, such as focusing on general healthcare workers without considering role-specific responsibilities or institutional factors [10–13]. Moreover, there is a lack of recent data reflecting IPC practices in the context of the updated national guidelines and post-COVID-19 strategies. Therefore, this study aimed to assess infection prevention practices and associated factors among healthcare professionals in public hospitals of West Gojjam Zone. The findings will support infection control programs by identifying gaps, evaluating current practices, and informing improvements in IPC implementation.

## Materials and methods

### Study design and settings

An institutional-based mixed cross-sectional study was conducted at 8 public hospitals in the West Gojjam zone, Amhara Region of Ethiopia, from March 10 to April 10, 2023. West Gojjam is one of the administrative zones in Amhara Regional State, Ethiopia, with the Town of Finote Selam**,** which is located 387 km away from Addis Ababa, the capital city of Ethiopia, and 176 km away from Bahir Dar, the capital city of Amhara Regional State. The total population of the zone was 2,611,925 based on the 2016/2017 fiscal year report [14]. There were seven primary public hospitals and one general hospital in this zone during data collection, namely, Finote Selam, Burie, Durbete, Merawi, Adet, Liben, Feres Bet, and newly added Dembecha, and there were more than 1265 healthcare professionals working on those hospitals.

### Source and study population

The source populations were all health care professionals (midwives, nurses, physicians, and laboratory professionals) who have common IP practice and role. The study populations were all health care professionals (midwives, nurses, physicians, and laboratory professionals) who have common IP practice and role and who work during data collection in all hospitals.

### Inclusion criteria

All healthcare professionals (midwives, nurses, physicians, and laboratory professionals) who have common IP practice and role and who have worked more than 6 months in all hospitals were included in the study.

### Sample size determination and sampling method for quantitative data

The sample size was determined by two population proportions and gives a total of 454 healthcare professionals. After the healthcare professionals were stratified by types of profession (general practitioner, laboratory, midwife, and nurse), a simple random sampling technique was used to select participants from all hospitals by considering proportional allocation.

### Sample size and sampling methods for qualitative data

For qualitative data, 8 key informants were purposefully selected (1 in each hospital) based on their role for IP practice. Key informants selected were five environmental health professionals working as IP focal, one nurse working as matron and IP focal, one monitoring and evaluation officer who was working as IP focal for more than three years, and one hospital manager who was working as IP focal in the hospital.

### Dependent variable

Infection prevention practice (good or poor)

### Independent variables

**Individual level factors** are those factors sociodemographic factors (gender, age, occupation, education level, work area, and experience) and behavioral factors (knowledge and attitude of healthcare professionals) of the individuals and,

**Institutional level factors**: availability of latrines, hand hygiene facilities, water, IPC guidelines, SOPs, training, availability of IPC supply (PPE. chemicals), IPC committee, supervision, and availability of IPC focal.

### Operational definitions

Infection prevention practice**:** infection prevention practices of healthcare professionals measured by 10 always/yes as 1 and sometimes/never/no as 0. Next, the summation of the 10 practice items was made. Then, the variable was recorded and dichotomized (good/poor).

**Good infection prevention practices:** For healthcare professionals who answered 70% of 10 practice questions [15].

**Knowledge:** Knowledge of infection prevention practices was measured using 13 yes/no knowledge assessment questions. Each correct answer “yes” scored “1” and “no” scored “0” points for each knowledge question. Then, the variable was recorded and dichotomized (1 as good, 0 as poor).

**Good knowledge of infection prevention practices:** for healthcare professionals who answered ≥ 70% of 13 knowledge questions correctly and 10 attitude questions [15].

**Attitude:** Attitude of infection prevention was measured by 10 questions of a five-point Likert scale from strongly agree to disagree, then the summation was made and recorded and dichotomized in to (good/poor).

**Good attitude of infection prevention practices:** For healthcare professionals who answered ≥70% of 10 attitude questions

**Healthcare professionals:** In this study, it includes professionals who have direct contact with infection prevention services and have the same role (doctors, nurses, midwives, and laboratories).

### Data collection tool and procedure for quantitative data

A structured, closed-ended questionnaire and an observational checklist were used to collect quantitative data. The tools were developed based on a review of national and international IPC guidelines and relevant literature. The questionnaire included six parts addressing socio-demographic characteristics, knowledge, attitude, individual practice-related factors, and institutional-related factors. The observational checklist focused on instrument processing and laundry service practices. Both tools were pre-tested on 5% of the sample size at Felege Hiwot Comprehensive Specialized Hospital to assess clarity and relevance. Necessary modifications were made based on the pretest. Data collection was carried out at public hospitals through a self-administered questionnaire, facilitated by eight trained data collectors (3 diploma nurses, 1 BSc nurse, and 4 BSc environmental health professionals). Verbal informed consent was obtained from participants after briefing them about the study purpose. For the qualitative component, data were collected through face-to-face, semi-structured interviews using an interview guide developed from IPC-related literature and national guidelines. The guide contained five open-ended questions aimed at exploring perceptions and institutional practices around IPC. Interviews were conducted by the principal investigator, audio-recorded with consent, transcribed in Amharic, and translated into English for analysis.

### Data quality assurance

Data quality was ensured by training data collectors for one day on the study objectives, tools, and data collection techniques. The principal investigator supervised the process and checked each completed questionnaire for completeness and consistency daily. For the qualitative data, credibility was supported through audio recordings, note-taking, repeated review of the recordings, and short feedback discussions with participants at the end of interviews to confirm the accuracy of key points discussed.

### Data management and analysis

Quantitative data were coded and entered into Epi-Data version 4.6, then exported to SPSS version 27 for analysis. Descriptive statistics, including frequencies and percentages, were used to summarize the data and were presented using tables and graphs. Bivariate and multivariate analyses were conducted using Generalized Estimating Equations (GEE) to account for the clustering effect within hospitals. Variables with a p-value <0.2 in bivariate analysis were included in the multivariate model, and those with a p-value <0.05 were considered statistically significant predictors. Qualitative data were analyzed manually using thematic content analysis. The principal investigator reviewed transcripts and audio recordings multiple times, coded the data, grouped codes into categories, and identified themes. The qualitative findings were then triangulated with the quantitative results to enhance interpretation and depth of understanding.

### Ethical consideration

Ethical clearance for this study was obtained from the Institutional Review Board (IRB) of the College of Medicine and Health Sciences, Bahir Dar University (Protocol Code: 720/2023; Date of Approval: March 6, 2023). A permission letter was obtained from the Amhara Public Health Institute (APHI) and from each participating health facility. Full information about the purpose and nature of the study was provided to all participants, and verbal informed consent was obtained before participation. Participation was voluntary, and participants were informed of their right to refuse or withdraw at any time. Confidentiality was ensured by excluding names and personal identifiers from the questionnaire.

## Result

### Socio-demographic characteristics of study participants

As summarized in Table 1, most participants were aged 25–30 years (60.8%), 59.9% were male, and 54.8% were married. Most were nurses (59.4%), followed by midwives (15.7%) and laboratory professionals (12.7%). Over three-fourths (77%) held a first degree, and 20% held a diploma. Participants were drawn from various departments, with the highest representation from outpatient departments (17.7%), followed by delivery/gyn ward (14.5%), and laboratory (12.2%). Over half (53.5%) had less than 5 years of work experience, while only 1.6% had over 15 years (Table 1).

**Table 1:** Socio-demographic characteristics of healthcare professionals participating in the study in West Gojjam Zone, June 2023 (n = 434).

Variables	Categories	Frequency	Percentage
Age of Participants	<25	36	8.3
	25–30	264	60.8
	31-35	109	25.1
	>35	25	5.8
Sex of Participants	Male	260	59.9
	Female	174	40.1
Marital status	Married	238	54.8
	Single	196	45.2
Profession of participants	Doctor	53	12.2
	Nurse	258	59.4
	Laboratory	55	12.7
	Midwife	68	15.7
Level of Education	Diploma	87	20
	First Degree	334	77
	Second Degree and Above	13	3
Working Department	OPD	77	17.7
	Surgical ward	37	8.5
	Delivery/Gyni	63	14.5
	Medical	42	9.7
	OR	33	7.6
	Laboratory	53	12.2
	Emergency	42	9.7
	Pediatrics	35	8.1
	Others	52	12
Work Experience in years	<5	232	53.5
	5–10	158	36.4
	11-15	37	8.5
	>15	7	1.6

Eight key informant interviews were conducted, involving five IPC focal persons, one matron, one hospital CEO, and one M&E officer. Of these, two were female and six were male. Educationally, two had master’s degrees in environmental health, three had bachelor’s degrees in environmental health, two held diplomas in the same field, and one had a BSc in Nursing.

### Knowledge of infection prevention and control guidelines and practices

As shown in Table 2, 75.3% of healthcare professionals knew their responsibility in IPC, and 96.8% correctly identified transmission mechanisms of infectious agents. While 70.3% knew the five critical hand hygiene moments, only 40.1% were aware of which parts of the IPC guideline were updated. Knowledge on healthcare waste segregation was high (82.9%), as was understanding of safety box capacity (80.6% chose three-quarters full). Most respondents (83.9%) identified chemicals used for high-level disinfection or sterilization, and 82.5% knew alcohol-based antiseptics are as effective as soap and water. However, 51.2% believed gloves could replace handwashing, and only 44.5% knew handwashing was required even before procedures without fluid contact. Knowledge gaps remain in guideline content, appropriate glove use, and preventive hygiene practices (Table 2).

**Table 2:** Knowledge of infection prevention and control (IPC) guidelines and practices among healthcare professionals (n = 434).

Variables	Response	Frequency	Percentage
Know IPC guideline is updated	Yes	198	45.6
	No	236	54.4
Knowing healthcare professionals’ responsibility	Yes	327	75.3
	No	107	24.7
Know transmission mechanisms of infectious agents	Yes	420	96.8
	No	14	3.2
Know critical movements of hand hygiene	Yes	305	70.3
	No	129	29.7
How many critical movements of hand hygiene (n = 305)	2	34	11.1
	5	238	78
	4	33	10.8
Know parts of the IPC guideline updated	Yes	174	40.1
	No	260	59.9
Know segregation of healthcare waste	Yes	360	82.9
	No	74	17.1
Know capacity of safety box	^1^/2 full	37	8.5
	3/4 full	350	80.6
	Full	38	8.76
	I don’t know	9	2.1
Know health hazard associated with healthcare waste	Yes	395	91
	No	39	9
Know gloves replace hand washing	Yes	212	48.8
	No	222	51.2
Know alcohol-based antiseptic for hand hygiene is as effective as soap and water	Yes	358	82.5
	No	76	17.5
Know wash hands before doing procedures that do not involve body fluid	Yes	193	44.5
	No	241	55.5
Know chemicals used for sterilization/high level disinfection	Yes	364	83.9
	No	70	16.1
Know availability of infection prevention guideline	Yes	245	56.5
	No	189	43.5

### Perceptions of infection prevention practices and occupational hazards

Table 3 summarizes healthcare workers’ attitudes toward infection prevention and occupational risk. The majority agreed or strongly agreed that IPC responsibility lies with healthcare workers (80%), and that healthcare environments expose workers to hazards (91%). Most recognized the need for accessible PPE (77.6%) and mandatory HBV vaccination (74.6%). However, misconceptions persisted; 85% disagreed or were neutral on recapping needles after use, indicating awareness of their risks. Hand hygiene at every patient contact was acknowledged by 67.2% of respondents. Overall, the findings show generally positive IPC perceptions with some gaps requiring targeted education (Table 3).

**Table 3:** Healthcare professionals’ perceptions of infection prevention practices and occupational risks (n = 438).

Variables	Frequency (%)				
	Strongly disagree	Disagree	Neutral	Agree	Strongly Agree
Soaking instruments with 0.5% Chlorine	83(19.1)	132(30.4)	12(2.8)	107(24.7)	100(23)
Responsibility of IPC	26(6.0)	49(11.3)	13(3.0)	155(35.7)	191(44)
Healthcare environments expose to occupational hazards	8(1.8)	16(3.7)	16(3.7)	159(36.6)	235(54.1)
PPE should be accessible in the working department	22(5.1)	56(2.9)	19(4.4)	135(31.1)	202(46.5)
Needles should be recapped after use	132(30.4)	237(54.6)	19(4.4)	21(4.8)	25(5.8)
Wearing face mask and eye goggles during procedures with aerosol production	18(4.1)	45(10.4)	11(2.5)	136(31.3)	224(51.6)
HBV vaccination for healthcare workers is mandatory	36(8.3)	55(12.7)	19(4.4)	100(23)	224(51.6)
Soaking soiled instruments with 0.5% chlorine solution	68(15.7)	106(24.4)	23(5.3)	93(21.4)	144(33.2)
Hepatitis B virus may be transmitted through biomedical wastes	6(1.4)	23(5.3)	8(1.8)	134(30.9)	263(60.6)
Hand hygiene in every patient contact	24(5.5)	94(21.7)	24(5.5)	87(20)	205(47.2)

### Hand hygiene, PPE use, and waste management practices

Table 4 summarizes healthcare workers’ compliance with hand hygiene, PPE use, and waste management practices. About 68% reported washing hands after patient contact, and 70.7% performed hand hygiene before and after glove use. Most (88.1%) consistently wore gloves during risky procedures, while 78.6% changed gloves between patients. Proper detergent use after patient contact was less consistent, with 48% always adhering. PPE use was reported as always by 65.9%, and 81.1% segregated healthcare waste properly. The use of safety guidelines was low, with only 23% always following them (Table 4).

**Table 4:** Hand hygiene practices, personal protective equipment use, and waste management behaviors among healthcare workers in public hospitals, West Gojjam Zone, Ethiopia, June 2023.

Variables	Response	Frequency	Percent
Washing your hands after patient contact	Yes	296	68.2
	No	138	31.8
Perform hand hygiene before and after glove	Yes	307	70.7
	No	127	29.3
Wear gloves during risky procedures	Always	382	88.1
	Sometimes	47	10.8
	Not at all	5	1.1
Wash your hands with proper detergent after contact with patients	Always	208	48
	Sometimes	213	49
	Not at all	13	3
Using proper PPE during professional practice	Always	286	65.9
	Sometimes	137	31.6
	Not at all	11	2.5
Monitoring working area waste management	Always	195	44.9
	Sometimes	160	36.9
	Not at all	79	18.2
Changing gloves between contacts with different patients	Always	341	78.6
	Sometimes	78	18
	Not at all	15	3.4
Use safety guideline/ manual at workplace	Always	100	23
	Sometimes	141	32.5
	Not at all	193	44.5
Segregating healthcare wastes according to their type	Yes	352	81.1
	No	82	18.9
Wearing gown in the hospital café	Yes	219	50.5
	No	215	49.5

### Infection prevention practice

The overall level of good infection prevention practice among healthcare professionals was low, with only 142 (32.7%) demonstrating good practice compared to 292 (67.3%) with poor practice. Notably, Merawi Hospital had the highest proportion of good IP practice 31 (56.4%), followed by Burie 26 (48.2%) and Durbete 17 (40.5%). In contrast, markedly low levels of good IP practice were observed in Adet 9 (16.4%), Finote Selam 20 (19.4%), and Dembecha 8 (25.8%) hospitals (S1 Fig).

### Availability and functionality of IPC facilities and practices

Table 5 presents the availability and functionality of infection prevention and control resources across public hospitals in West Gojjam Zone. Most hospitals (72.8%) had hand hygiene facilities available, although only 47.5% of these were functional. Water availability varied, with 35% of hospitals reporting continuous access, while the remainder had intermittent supply. Waste bin availability and staff training based on updated guidelines were each reported in about half of the hospitals (48.8% and 48.8%, respectively). Availability of IPC supplies was reported in 61.5% of hospitals. Latrine availability was higher, at 76.3%. Only 36.4% of hospitals had IPC guidelines available on-site. All hospitals had an IPC focal person, and 58.8% had a functional IPC committee. However, refresher training in the last six months was low, at 12.7%, and supervision based on guidelines was reported in 54.8% of facilities (Table 5).

**Table 5:** Availability and functionality of IPC facilities and practices in public hospitals, West Gojjam Zone, Ethiopia, June 2023. (n = 434).

Variables	Categories	Frequency	Percent
Hand Hygiene Facility Availability	No	118	27.2
	Yes	316	72.8
Functional HHF	No	228	52.5
	Yes	206	47.5
Availability of water	Once per week	173	39.9
	Twice per week	109	25.1
	all time	152	35.0
Availability of waste bins	No	222	51.2
	Yes	212	48.8
Training based on the new guideline	No	222	51.2
	Yes	212	48.8
Availability of IPC supplies	No	167	38.5
	Yes	267	61.5
Availability of latrines	No	103	23.7
	Yes	331	76.3
Availability of guideline	No	276	63.6
	Yes	158	36.4
IPC focal for each hospital	Yes	434	100.0
Functional IPC committee	No	179	41.2
	Yes	255	58.8
Refresher training in the last six months	No	379	87.3
	Yes	55	12.7
supervision based on the guideline	No	196	45.2
	Yes	238	54.8

### Instrument processing compliance

As presented in Table 6, Burie, Finote Selam, and Merawi hospitals each complied with 4 out of 6 assessed standards (66.7%), followed by Adet and Durbete hospitals with scores of 3 (50.0%). Dembecha, F/Bet, and Liben hospitals reported the lowest scores, fulfilling only 2 standards (33.3%). Adequate space for instrument processing was the most consistently met criterion, reported by 7 of the 8 hospitals (87.5%). SOP availability and cleaning before soaking was fulfilled in 4 hospitals (50%) each. Spaulding classification was applied in only 3 hospitals (37.5%). Soaking soiled instruments in chlorine was practiced in 4 hospitals (50%). None of the hospitals had chemicals available for high-level disinfection or sterilization, reflecting a uniform gap across all facilities (Table 6).

**Table 6:** Instrument processing compliance scores across public hospitals in West Gojjam Zone, Ethiopia, June 2023.

Hospital	Soaked in Chlorine	SOP Available	Cleaned Before Soaking	Spaulding Classification Used	Adequate Space	Chemicals Available	Total Score (0–6)	% Compliance
Adet	No	No	Yes	No	Yes	No	3	50.0
Burie	No	Yes	Yes	Yes	Yes	No	4	66.7
Dembecha	Yes	No	No	No	Yes	No	2	33.3
Durbete	Yes	Yes	No	No	Yes	No	3	50.0
F/Bet	Yes	No	No	No	Yes	No	2	33.3
Finote Selam	No	Yes	Yes	Yes	No	No	4	66.7
Liben	Yes	No	No	No	Yes	No	2	33.3
Merawi	No	Yes	Yes	No	Yes	No	4	66.7

***Note: “Yes” = practice performed; “No” = not performed. Total score out of 6. % Compliance = (Score ÷ 6) × 100.***

It is supported by key informants; challenges included the absence of updated IPC guidelines and difficulty obtaining chemicals for high-level disinfection and instrument processing. *‘A 26-year-old male key informant said the ministry of health updated the IPC guidelines and prohibited chlorine and changed to other chemicals, but those chemicals are not affordable and easily available at the market, for example, hydrogen per oxide greater than 7% concentration.”*

### Laundry service compliance

As shown in Table 7, Finote Selam Hospital demonstrated full compliance with all five assessed laundry service standards (100%), followed by Burie Hospital with a score of 4 out of 5 (80%). Adet and Merawi hospitals each fulfilled 3 standards (60%). The lowest compliance was observed at Dembecha and F/Bet hospitals, each scoring only 1 (20%). The most commonly met criteria across hospitals were the use of separate carts for clean and soiled linens and staff use of appropriate personal protective equipment (PPE), each implemented in 6 out of 8 hospitals (75%). In contrast, soaking soiled linens in chlorine and availability of SOPs were each practiced by only 2 hospitals (25%), indicating significant gaps in infection prevention protocols related to laundry handling (Table 7).

**Table 7:** Compliance with standard laundry service practices across public hospitals in West Gojjam Zone, Ethiopia, June 2023.

Hospital	Soaked in Chlorine (15–30 min)	SOP Available	Separate Cart for Cleaned and Soiled Linens	Staff Wore Appropriate PPE	Staff Trained by New Guideline	Total Score (0–5)	% Compliance
Adet	No	No	Yes	Yes	Yes	3	60.0
Burie	Yes	No	Yes	Yes	Yes	4	80.0
Dembecha	No	No	Yes	No	No	1	20.0
Durbete	No	Yes	No	Yes	No	2	40.0
F/Bet	No	No	No	Yes	No	1	20.0
Finote Selam	Yes	Yes	Yes	Yes	Yes	5	100.0
Liben	No	No	No	Yes	Yes	2	40.0
Merawi	No	Yes	Yes	Yes	No	3	60.0

***Note: “Yes” = practice implemented; “No” = not implemented. Total score is out of 5. % Compliance = (Score ÷ 5) × 100.***

### Predictors of infection prevention practice

In the bivariate generalized estimation equation (GEE) analysis, variables with a p-value less than 0.2, specifically knowledge, attitude, the midwifery profession, and employment in certain hospitals (Adet, Dembecha, Durbete, Feres Bet, Finote Selam, and Liben), were identified as candidates for multivariate analysis. The multivariate results showed that healthcare professionals with good knowledge were nearly twice as likely to demonstrate good infection prevention (IP) practices compared to those with poor knowledge (AOR = 1.95, 95% CI: 1.19–3.19). Similarly, those with a positive attitude had nearly double the odds of good IP practice compared to those with a poor attitude (AOR = 1.96, 95% CI: 1.24–3.12). These findings were reinforced by qualitative evidence. A 25-year-old IP focal person noted that “most healthcare professionals don’t care about IP; even if they are knowledgeable, they dump infectious waste on the ground and ignore hand hygiene.” In contrast, a 36-year-old key informant stated, “attitude is not a problem in our hospital; if all necessary supplies and facilities are fulfilled, the professionals can apply proper IPC practices.” Profession was also a significant factor: midwives were 53% less likely to demonstrate good IP practice compared to nurses (AOR = 0.47, 95% CI: 0.24–0.92). Marked variation was observed across hospitals. Compared to Merawi Hospital, the odds of good IP practice were significantly lower in Adet (AOR = 0.18, 95% CI: 0.08–0.45), Dembecha (AOR = 0.26, 95% CI: 0.09–0.76), Durbete (AOR = 0.44, 95% CI: 0.20–0.99), Feres Bet (AOR = 0.35, 95% CI: 0.15–0.83), Finote Selam (AOR = 0.18, 95% CI: 0.08–0.39), and Liben (AOR = 0.40, 95% CI: 0.18–0.95). (Table 8).

**Table 8:** Factors associated with infection prevention practice among healthcare professionals in public hospitals, West Gojjam Zone, Ethiopia (n = 434).

Variables	Category	IP Practice		Parameter estimates		
		Good	Poor	COR 95%CI	AOR 95%CI	P-Value
Hospitals	Merawi	31	24	1	1	
	Adet	9	46	0.15(0.06-0.37)	0.18(0.08-0.45)	0.001
	Burie	26	28	0.72(0.34-1.53)	0.50(0.23-1.10)	0.085
	Dembecha	8	23	0.27(0.103-0.71)	0.26(0.09−.762)	0.014
	Durbete	17	25	0.53(0.23-1.19)	0.44(0.196-0.99)	0.048
	F/Bet	14	35	0.31(0.12-0.70)	0.35(0.15-0.83)	0.017
	Finote Selam	20	83	0.19(0.09-0.38)	0.181(0.08-0.39)	0.001
	Liben	17	28	0.47(0.21-1.05)	0.40(0.180-0.95)	0.026
Marital status	Single	57	139	1	1	
	Married	85	153	1.35(0.902-2.034)	0.64(0.40-1.01)	0.058
Profession	GP	15	38	1	1	
	Laboratory	19	36	1.09(0.590-2.00)	1.06(0.55-2.06)	0.860
	Midwife	14	54	2.21(1.17-4.19)	0.47(0.24-0.92)	0.028
	Nurse	94	164	1.45(0.76-2.78)	1.05(0.51-2.2)	0.887
Knowledge	Poor	44	152	1	1	
	Good	98	140	2.42 (1.58-3.69)	1.95(1.19-3.19)	0.008
Attitude	Poor	56	178	1	1	
	Good	86	114	2.4 (1.59-0.48)	1.96 (1.24-3.12)	0.004
HH Facility Availability	No	42	76	1	1	
	Yes	100	216	0.84(0.537 - 1.31)	1.6(0.27-9.39)	0.603
Functional HHF	No	82	146	1	1	
	Yes	60	146	0.73(0.49 - 1.096)	1.35(0.33-5.49)	0.672
Availability of water	Once per week	51	122	1	1	
	Twice per week	57	52	2.6 (0.878-6.199)	2.14(0.70-6.52)	0.183
	All time	34	118	0.69(0.42-1.14)	0.58(.059-5.76)	0.646
Training based on new guideline	No	55	157	1	1	
	Yes	87	155	1.6 (1.22-2.78)	1.49(0.47- 4.73)	0.496
IPC Supply	No	56	111			
	Yes	86	181	0.94 (0.62-1.42)	0.47(0.21-1.07)	0.073
Latrine Availability	No	20	28	1	1	
	Yes	122	209	0.82 (0.41-1.25)	1.10 (0.46-2.55)	0.818
IPC Committee	No	45	134	1	1	
	Yes	97	158	1.83 (1.2-2.78)	0.80 (0.42-1.52)	0.500

COR = crude odds ratio, AOR = adjusted odds ratio, CI = confidence interval, 1 = reference

### Challenges of Infection Prevention (IP) practice: insights from key informant interviews

Nearly all key informants agreed that the practice of infection prevention (IP) was influenced by multiple factors. From the interview analysis, six key themes emerged, broadly categorized into institutional factors (including infrastructure, IPC supplies, training, management systems, and guidelines) and healthcare professional-related factors (notably attitude and commitment).

### IP infrastructure and supplies

One major barrier to effective IP practice in healthcare facilities was the inadequacy of essential infrastructure, particularly the availability of water, latrines, and functional hand hygiene stations. Across the seven hospitals included in the interviews, hand hygiene facilities and latrines were reportedly available; however, in almost all cases, the hand hygiene stations were non-functional. Most hospitals also face poor water installation systems and recurrent water shortages. The lack of clean and consistent water supply was consistently cited as a serious problem by key informants. Due to this challenge, healthcare professionals were unable to practice hand hygiene in accordance with WHO guidelines. Additionally, the shortage of water had a direct negative impact on laundry services and instrument processing, further compromising infection prevention measures in these facilities.

*‘’A 37-year-old male key informant said that our hospital got water from the municipality twice per week as the community and the installations of the pipe system were not properly functional; at least we tried to reinstall the pipe system to decrease the problems related to water’’*. ’ *Another 36-year-old male key informant said that we built a deep well and we have enough water in our hospital even though we are serving the surrounding community, but our hospital had a shortage of latrines, and one latrine was used for more than 100 people per day.’’*

For healthcare facilities to maintain good IP practices, there must be 24-hour access to water, sufficient and functional hand hygiene facilities in service areas, and adequate latrines with hand hygiene stations located within a 5-meter radius for both patients and staff. To improve IP practices, healthcare facilities should also be equipped with adequate supplies (PPE, detergents, disinfectants, bins, etc.); however, there were shortages of IP supplies in the hospitals assessed. For example, some hospitals lacked standardized color-coded bins, high-level disinfectants, and safety boxes. *‘A 37-key informant said that even if we had money to buy color-coded bins, there was no access at the market, and safety boxes* *are not available totally at the market’. ‘Another 26-year-old key informant said the Ministry of Health updated the IP guidelines and prohibited chlorine and changed to other chemicals, but those chemicals are not affordable and easily available at the market; for example, it is difficult to get safety boxes, color-coded waste bins, and hydrogen peroxide concentrations greater than 7%.’’*

### IP training

This is a barrier that hinders IP practice, as agreed upon by almost all key informants. IP training is mandatory for every healthcare professional to introduce policies, guidelines, and SOPs, especially during new recruitments or when guidelines are updated. The guideline also recommends that refresher training be provided every six months. However, based on these interviews and observations, all key informants reported that although the IP guidelines had been updated, healthcare professionals had not received training, and the guidelines were not available in those hospitals. *“A 26-year-old key informant said that I heard the guideline was updated in 2019, but no one in our hospital didn’t get training, including me.” ‘Another 36-year-old key informant said that we were lucky our 15 healthcare professionals got training by NGOs in our hospital (World Vision IPAS).’‘*

The IP guideline must be available in all wards. Based on the updated guideline, healthcare professionals or those responsible for IP in the hospital must prepare SOPs for waste management, instrument processing, laundry service, housekeeping, injection safety, and ensure their availability in service areas. Healthcare professionals can then act according to these SOPs and keep themselves updated.

### Hospital management system

Weak management in hospitals undermines IP practice. Hospital management should form an IP committee and appoint an IPC focal person, and they should review and supervise activities at least monthly based on the guidelines. The IPC focal person should report to management and supervise wards daily. However, in these hospitals, management was poor; although there was an IPC focal person in all hospitals, only two hospitals had functional IPC committees. *‘’A 34-year-old key informant said that there was a disease prevention team that actively followed IP activities and prepared refresher training before, but now there is no such team, and IPC becomes an under-quality team, and no one knows the status of IP in our hospital.’‘*

*Another 39-year-old male key informant said that weekly Friday afternoon we had a cleaning campaign with all healthcare professionals, for those who participate, there is punishment.”*

To increase IP practice in healthcare facilities, hospital management must organize cleaning campaigns hospital-wide and in each ward according to activities and establish reward mechanisms for those who perform better.

### IP guidelines

Except for two key informants, all others agreed there was a shortage of IPC guidelines in their hospitals. To improve IP practice, IPC guidelines and SOPs should be available in every department. SOPs must be prepared based on the existing guidelines. ‘A 35-year-old key informant said *we have got only one copy of the updated guideline from the Ministry of Health, and we have given IP training for healthcare professionals. To reach the guidelines to healthcare professionals, we created a Telegram channel, and everyone can access it.’.*

### Healthcare professionals attitude and commitment

Healthcare professionals’ attitude and commitment have a significant impact on IP practice. Even if healthcare workers are knowledgeable and IPC supplies are sufficient, IP practice will not improve without a positive attitude and strong commitment. *‘A 25-year-old key informant said that most healthcare professionals don’t care about IP; even if they are knowledgeable, they dump infectious waste in the ground and the hand hygiene facility.’*

*‘Another 36-year-old key informant said attitude is not a problem in our hospital; if we fulfill all necessary supplies and facilities, healthcare professionals can apply proper IP practice’.*

To improve the attitude of healthcare professionals, hospital management should act to fulfill necessary facilities, guidelines, and SOPs, and prepare onsite training to enhance their knowledge and sense of belonging. Generally, based on these key informants, many challenges hinder healthcare professionals from applying good IP practices. Among them, a shortage of budget or lack of any budget for IP practice was a major challenge in almost all hospitals. However, one key informant said, “That budget is not the real problem; the big problem is the professionals who are working on IP.” Another challenge was a shortage of water, especially in six hospitals. Water availability from the municipality was once or twice a week, similar to the community. A 25-year-old key informant said, “Our hospital gets water once per week from the municipality if there is electricity. This problem is especially severe in laundry, minor OR, and delivery services. We buy water for those services at 7 birr per 20 liters.”

Guidelines were lacking in six hospitals, and poor attitudes among healthcare professionals and IP personnel, along with a lack of concern from the Ministry of Health towards hospital management, posed major challenges. One key informant stated, “The responsible bodies from Federal to Regional levels and down to ward IP have poor attitude and commitment; they didn’t even try to provide guidelines or prepare trainings, especially for IPC focal persons, nor did they supervise whether updated IPC guidelines were implemented.”

## Discussion

The primary objective of infection prevention and control is to reduce the risk of infection in healthcare settings and support the delivery of quality care. Achieving this requires healthcare professionals to possess both good knowledge and a positive attitude toward infection prevention. This study revealed that the overall magnitude of good IPC practice among healthcare professionals was 32.7%, consistent with a study conducted in the Bale Zone (36%) [16]. The similarity may be due to comparable definitions of good IPC practice and the professional mix of respondents. However, this finding is lower than those reported in recent studies, including a systematic review and meta-analysis in Ethiopia reporting a pooled IPC practice of 55% [8], a study conducted in northeastern Ethiopia with 53.7% adherence [17], and a study from Addis Ababa reporting 36.5% standard precaution compliance among healthcare professionals [18]. Differences in cutoff scores, sample size, and the scale of healthcare institutions studied may explain this variation. On the other hand, our result is higher than a study from Northeastern Ethiopia (19%) [17], possibly due to differences in scoring systems, study setting, or facility type.

However, compared to international contexts, our findings remain lower than more recent studies. For instance, in Cameroon’s Fako Division, approximately 64.5% of healthcare workers demonstrated compliance with IPC standard precautions [19]. In Ghana, during the COVID-19 response, healthcare workers exhibited high compliance rates, 88.4% for hand hygiene and 90.6% for PPE use [20], likely reflecting disparities in resources, healthcare infrastructure, and institutional commitment. These figures underscore that IPC adherence can exceed 80% in comparable LMIC settings, highlighting a substantial gap in our local infection prevention practices.

Significant variations in IPC practice were observed across hospitals, ranging from 56.4% in Merawi to just 16.4% in Adet. Generalized estimating equation (GEE) analysis confirmed statistically significant differences. Qualitative data supported this, with informants citing differences in availability of training (only Merawi and Finote Selam had staff trained on the updated IPC guideline), presence of functional IPC committees, infrastructure, and daily water access as key differentiators. Instrument processing compliance was highest in Burie, Finote Selam, and Merawi (66.7%), but notably low in Durbete, Feres Bet, and Liben (16.7%). Qualitative findings corroborated these results, highlighting the absence of SOPs, limited availability of disinfectants, and lack of processing equipment in poorly performing hospitals.

Similarly, the quality of laundry services varied greatly. Finote Selam (100%) and Burie (80%) scored highest, attributed to the presence of SOPs, training for laundry staff, and consistent water access. Key informants in poorly performing facilities cited shortages of water, budget constraints, and lack of training as primary barriers.

Knowledge emerged as a significant predictor: those with good knowledge were nearly twice as likely to practice good IPC (AOR = 1.95, 95% CI: 1.19–3.19). However, only 54% of healthcare workers demonstrated adequate knowledge, likely due to the lack of access to the updated IPC guidelines and inadequate refresher training. As supported by other studies [21,22], access to updated guidelines and training plays a crucial role in enhancing IPC compliance.

Attitude also significantly influenced IPC practice (AOR = 1.96, 95% CI: 1.24–3.12), aligning with studies from North Shewa, Addis Ababa, and Trinidad and Tobago [10,23,24]. Qualitative findings further supported this, with key informants frequently citing poor attitude and commitment among staff as barriers to implementation, even when supplies were available. Conversely, one informant noted that if facilities and guidelines were adequately provided, staff attitudes tended to improve. Midwives were 53% less likely than nurses to follow IPC protocols, consistent with findings from Debre Markos and West Arsi [11,12]. This could be due to higher workloads and the urgent nature of maternity services, which often limit time for preventive measures. Access to clean water was another major determinant. Only 35% of participants reported daily water availability. Most hospitals receive water from the municipality only once or twice weekly, and poor plumbing further hindered access. This challenge aligns with findings from Addis Ababa and Hawassa [10, 21] and was emphasized by key informants, who reported that even basic hand hygiene could not be consistently performed due to water shortages.

In terms of training, although 48.8% of professionals had received IPC training, only two hospitals had copies of the updated guidelines. The absence of regular refresher sessions has hindered effective practice. While training showed significance in bivariate analysis, it was not statistically significant in multivariate analysis. This may reflect the lack of structured, standardized training programs based on updated content. Key informants acknowledged that although the guideline was revised in 2019, most staff had not been trained on the changes. In resource-limited settings, training programs should prioritize on-site modular refreshers, use low-cost printed SOPs, and train IPC focal persons who can cascade knowledge through peer-led sessions. Integration with routine hospital quality improvement activities and alignment with existing mentorship or supervision systems can also enhance implementation without requiring additional budget allocations. Availability of IPC supplies was another challenge, only 61.5% of professionals had adequate materials. Informants reported that items like high-level disinfectants and color-coded bins were either unavailable or too expensive. Similar findings have been reported in Ethiopia and Nigeria [11,25,26].

Budget constraints were highlighted by almost all key informants. Effective IPC requires investment in infrastructure, supplies, and training, yet it is often not prioritized. This reflects broader issues of underfunding, echoed in studies from Nigeria, LMICs, and WHO reports [27–29]. In summary, this study shows that despite lessons from COVID-19, IPC practices in the study hospitals remain suboptimal. Significant investments in training, infrastructure, and policy enforcement are needed to improve outcomes.

### Limitations of the study

In quantitative data, there might be social desirability bias regarding practice towards infection prevention. The other limitation of this study is that generalizability to all types of healthcare facilities will be difficult as it was concentrated only on hospitals. The study didn’t assess all parts of IPC practice.

## Conclusion

In this study, healthcare professionals’ infection prevention and control practice was found to be poor, with significant associations identified between IPC practice and factors such as attitude, knowledge, professional category, and affiliation with hospitals like Adet, Dembecha, Durbete, Feres Bet, Finote Selam, and Liben. Major challenges included lack of training, insufficient budget, water shortages, and weak institutional commitment. To address these gaps, hospital administrations should strengthen regular IPC training programs by utilizing in-house experts and integrating them into routine operations. Policymakers are encouraged to establish dedicated IPC budget lines and prioritize IPC within hospital financing and supervision systems. Strengthening partnerships with regional health authorities, NGOs, and academic institutions can provide additional technical and financial support. Ensuring functional IPC committees, consistent water supply, updated guidelines, and clear standard operating procedures across departments are essential for improving IPC practice in resource-limited settings.

## Supporting information

S1 Fig Infection Prevention Practices of Healthcare Professionals by Public Hospital in West Gojjam Zone, June 2023 (n = 434). (TIFF)

## Abbreviations

AOR: adjusted odds ratio
AMR: antimicrobial resistance
CEO: Chief Executive Officer
CDC: Communicable Diseases Control
CI: Confidence Interval
COR: crude odds ratio
HH: hand hygiene
HHF: hand hygiene facility
HAIs: healthcare-associated infections
IPC: infection prevention and control
PPE: Personal protective equipment
SOPs: standard operating procedures
WHO: World Health Organization.

## References

[1] World Health Organization. Global report on infection prevention and control. Geneva: World Health Organization; 2022. Available from: https://www.who.int/publications/i/item/9789240051164

[2] Ministry of Health Ethiopia. Ethiopia National Infection Prevention and Control Policy. Addis Ababa: Ministry of Health Ethiopia; 2022. Available from: https://resolvetosavelives.org/resources/ethiopia-national-infection-prevention-and-control-policy/

[3] Tomczyk S, Twyman A, de Kraker MEA, Coutinho Rehse AP, Tartari E, Toledo JP, et al. The first WHO global survey on infection prevention and control in health-care facilities. Lancet Infect Dis. 2022;22(6):845–56. doi: 10.1016/S1473-3099(21)00809-4 35202599 PMC9132775

[4] Ling ML, Apisarnthanarak A, Madriaga G. The Burden of Healthcare-Associated Infections in Southeast Asia: A Systematic Literature Review and Meta-analysis. Clin Infect Dis. 2015;60(11):1690–9. doi: 10.1093/cid/civ095 25676799

[5] Balakrishnan VS. WHO’s first global infection prevention and control report. Lancet Infect Dis. 2022;22(8):1122. doi: 10.1016/S1473-3099(22)00459-5 35870460 PMC9299724

[6] Barrera-Cancedda AE, Riman KA, Shinnick JE, Buttenheim AM. Implementation strategies for infection prevention and control promotion for nurses in Sub-Saharan Africa: a systematic review. Implement Sci. 2019;14(1):111. doi: 10.1186/s13012-019-0958-3 31888673 PMC6937686

[7] Tartari E, Hopman J, Allegranzi B, Gao B, Widmer A, Cheng VC-C, et al. Perceived challenges of COVID-19 infection prevention and control preparedness: A multinational survey. J Glob Antimicrob Resist. 2020;22:779–81. doi: 10.1016/j.jgar.2020.07.002 32659504 PMC7351656

[8] Sahiledengle B, Tekalegn Y, Woldeyohannes D. The critical role of infection prevention overlooked in Ethiopia, only one-half of health-care workers had safe practice: A systematic review and meta-analysis. PLoS One. 2021;16(1):e0245469. doi: 10.1371/journal.pone.0245469 33444417 PMC7808611

[9] Moh E. Advanced and special setting infection prevention and control (IPC). 2019.

[10] Angaw DA, Gezie LD, Dachew BA. Standard precaution practice and associated factors among health professionals working in Addis Ababa government hospitals, Ethiopia: a cross-sectional study using multilevel analysis. BMJ Open. 2019;9(10):e030784. doi: 10.1136/bmjopen-2019-030784 31615798 PMC6797290

[11] Desta M, Ayenew T, Sitotaw N, Tegegne N, Dires M, Getie M. Knowledge, practice and associated factors of infection prevention among healthcare workers in Debre Markos referral hospital, Northwest Ethiopia. BMC Health Serv Res. 2018;18(1):465. doi: 10.1186/s12913-018-3277-5 29914477 PMC6006704

[12] Geberemariyam BS, Donka GM, Wordofa B. Assessment of knowledge and practices of healthcare workers towards infection prevention and associated factors in healthcare facilities of West Arsi District, Southeast Ethiopia: a facility-based cross-sectional study. Arch Public Health. 2018;76:69. doi: 10.1186/s13690-018-0314-0 30455882 PMC6231270

[13] Markos T, Sinkie SO, Garedew MG. Compliance with infection prevention practices and associated factors among health professionals in the public hospitals of Kembata Tembaro Zone, south Ethiopia. Health Science Journal. 2021.

[14] Munea AM, Alene GD, Debelew GT, Sibhat KA. Socio-cultural context of adolescent sexuality and youth friendly service intervention in West Gojjam Zone, Northwest Ethiopia: a qualitative study. BMC Public Health. 2022;22(1):281. doi: 10.1186/s12889-022-12699-8 35148701 PMC8840675

[15] Gezie H, Leta E, Admasu F, Gedamu S, Dires A, Goshiye D. Health care workers knowledge, attitude and practice towards hospital acquired infection prevention at Dessie referral hospital, Northeast Ethiopia. Clin J Nurs Care Pract. 2019;3(1):059–63. doi: 10.29328/journal.cjncp.1001019

[16] Zenbaba D, Sahiledengle B, Bogale D. Practices of Healthcare Workers regarding Infection Prevention in Bale Zone Hospitals, Southeast Ethiopia. Advances in Public Health. 2020;2020:1–7. doi: 10.1155/2020/4198081

[17] Keleb A, Lingerew M, Ademas A, Berihun G, Sisay T, Adane M. The magnitude of standard precautions practice and its associated factors among healthcare workers in governmental hospitals of northeastern Ethiopia. Front Health Serv. 2023;3:1071517. doi: 10.3389/frhs.2023.1071517 37033899 PMC10073742

[18] Senbato B, Alemayehu Y, Gebeyehu A, Adamu A, Mekonnen A. Standard precaution practice and associated factors among healthcare workers in public hospitals of Addis Ababa, Ethiopia. SAGE Open Med. 2024;12. doi: 10.1177/20503121241231148

[19] Tsague MS, Mbapah LT, Teuwafeu DG, Jabbossung FE, Etaka ST, Ngunjoh NF, et al. Compliance with infection prevention and control standard precautions and associated factors among healthcare workers in four health facilities in Fako division, Cameroon. BMC Health Serv Res. 2025;25(1):596. doi: 10.1186/s12913-025-12594-z 40275306 PMC12023434

[20] Osei S, Frimpong-Manso S, Nguah SB, Atobrah D, Effah K, Owusu-Ansah FE, et al. Healthcare workers’ compliance with infection prevention and control practices: a cross-sectional study during COVID-19 in Ghana. Trop Med Int Health. 2021;26(3):358–66.

[21] Bekele T, Ashenaf T, Ermias A, Arega Sadore A. Compliance with standard safety precautions and associated factors among health care workers in Hawassa University comprehensive, specialized hospital, Southern Ethiopia. PLoS One. 2020;15(10):e0239744. doi: 10.1371/journal.pone.0239744 33057417 PMC7561127

[22] Thazha SK, Cruz JP, Alquwez N, Scaria B, Rengan SS, Almazan JU. Infection prevention and control awareness, attitudes, and practices among healthcare professionals in South India. J Infect Dev Ctries. 2022;16(4):659–67. doi: 10.3855/jidc.14746 35544628

[23] Jemal K, Gashaw K, Kinati T, Bedada W, Getahun B. Clean and Safe Healthcare Environment: Knowledge, Attitude, and Practice of Infection Prevention and Control among Health Workforce at North Showa Zone Oromiya Region. J Environ Public Health. 2020;2020:6021870. doi: 10.1155/2020/6021870 33178291 PMC7648680

[24] Unakal CG, Nathaniel A, Keagan B, Alexandria B, Lauralee B, Varun C, et al. Assessment of knowledge, attitudes, and practices towards infection prevention among healthcare workers in Trinidad and Tobago. Int J Community Med Public Health. 2017;4(7):2240. doi: 10.18203/2394-6040.ijcmph20172813

[25] Beyamo A, Dodicho T, Facha W. Compliance with standard precaution practices and associated factors among health care workers in Dawuro Zone, South West Ethiopia, cross sectional study. BMC Health Serv Res. 2019;19(1):381. doi: 10.1186/s12913-019-4172-4 31196064 PMC6567427

[26] Brisibe S, Ordinioha B, Gbeneolol PK. Knowledge, attitude, and infection control practices of two tertiary hospitals in Port-Harcourt, Nigeria. Niger J Clin Pract. 2014;17(6):691–5. doi: 10.4103/1119-3077.144379 25385903

[27] Dramowski A, Bekker A, Anugulruengkitt S, Bayani O, Martins Gonçalves F, Naizgi M. Keeping it real: Infection prevention and control problems and solutions in low- and middle-income countries. Paediatric Infect Dis J. 2022;41(3S):S36–9. 35134038 10.1097/INF.0000000000003319 PMC8815840

[28] Loftus MJ, Guitart C, Tartari E, Stewardson AJ, Amer F, Bellissimo-Rodrigues F, et al. Hand hygiene in low- and middle-income countries. Int J Infect Dis. 2019;86:25–30. doi: 10.1016/j.ijid.2019.06.002 31189085

[29] Lowe H, Woodd S, Lange IL, Janjanin S, Barnet J, Graham W. Challenges and opportunities for infection prevention and control in hospitals in conflict-affected settings: a qualitative study. Confl Health. 2021;15(1):94. doi: 10.1186/s13031-021-00428-8 34930364 PMC8686079

