# Supplementary figures and images for: Infection prevention practices and associated factors among healthcare professionals in West Gojjam Zone public Hospitals Northwest Ethiopia, 2023
Source: PLoS One. 2026 Jan 30;21(1):e0338621. doi: 10.1371/journal.pone.0338621 (PMC12858006; doi:10.1371/journal.pone.0338621)

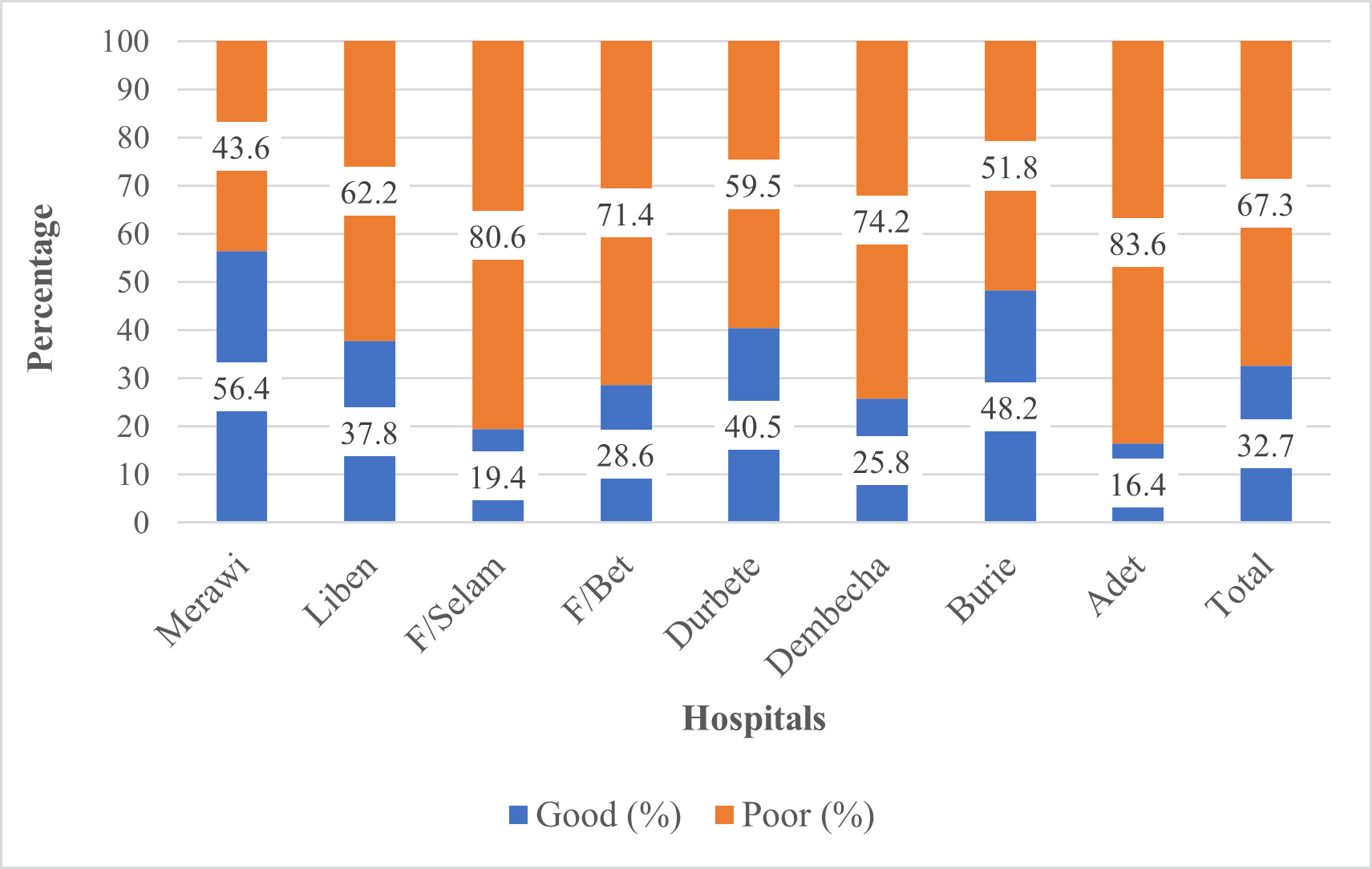

Supplement: S1 Fig — (TIFF) [file pone.0338621.s001.tiff]
